# pH-regulated hydrothermal synthesis and characterization of Sb_4_O_5_X_2_ (X = Br/Cl) and its use for the dye degradation of methyl orange both with and without light illumination†

**DOI:** 10.1039/d2ra01215d

**Published:** 2022-03-15

**Authors:** Sayantani Paul, Bibaswan Sen, Nirman Chakraborty, Sangita Das, Swastik Mondal, Asoke P. Chattopadhyay, Sk Imran Ali

**Affiliations:** Department of Chemistry, University of Kalyani Nadia West Bengal India skimranchem18@klyuniv.ac.in; CSIR-Central Glass and Ceramic Research Institute Jadavpur Kolkata West Bengal India

## Abstract

A pH-regulated hydrothermal synthesis method was employed to synthesize Sb_4_O_5_Br_2_ and Sb_4_O_5_Cl_2_ crystallites. Characterization is done by single crystal X-ray diffraction, powder X-ray diffraction, infra-red spectroscopy, scanning electron microscopy and DFT studies. The compounds crystallize in monoclinic symmetry with a *P*2_1_/*c* space group. Complete structural analysis of the Sb_4_O_5_Br_2_ compound by using single crystal X-ray diffraction data is performed for the first time and a comparative study with Sb_4_O_5_Cl_2_ is also discussed. The SEM study reveals that the surface morphology changes with the variation of pH for bromide compounds, whereas pH change does not affect the morphology of the chloride analogues. Electronic band structures of the synthesized oxyhalides were investigated in order to understand their catalytic effects in the dye degradation reactions in dark as well as sunlight conditions.

## Abstract 2

A pH-regulated hydrothermal synthesis method was employed to synthesize Sb_4_O_5_Br_2_ and Sb_4_O_5_Cl_2_ crystallites.

## Graphical abstract

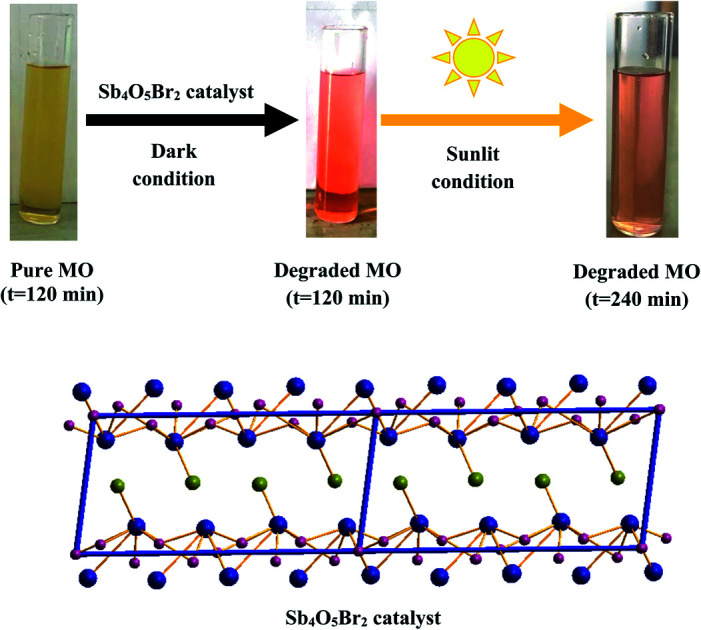

## Introduction

Photocatalytic materials have attracted considerable attention in recent years due to their potential use as catalysts in light-induced energy harvesting reactions and applications in waste water treatment, hydrogen fuel synthesis and so on.^1^ The presence of organic dye pollutants in the environment causes severe harm to public life, emphasizing the need to develop new types of photocatalysts to degrade organic dye contaminants in waste water, which will eventually aid the textile, printing, and dyeing industries.^2,3^

Efficiency of catalytic activities can be controlled by modifying the morphologies of catalyst-particles and by tuning light absorption abilities and band gaps of catalysts.^1,4–10^ The change of reaction conditions, *e.g.*, pH regulated synthesis, plays an important role for designing these kinds of catalyst materials depending on the reaction conditions they may form different kinds of phases, sizes, shapes and surfaces.^11^ As far as light absorption is concerned, much of the solar radiation can be harvested by narrowing the band gap of catalysts to allow the valence electron to be excited by lower energy photons. A photocatalytic reaction can be defined as a chemical reaction in which photons interact with a semiconducting material known as a photocatalyst and facilitate the reaction.^12^ Plenty of metal oxides (*e.g.*, TiO_2_, ZnO, MnO_2_, CeO_2_, CdO *etc.*), metal halide perovskites (MHP) (*e.g.*, CsPbX_3_ (X = I, Br, Cl)) and polymers have been established as photocatalysts so far.^13^ Recently, oxyhalide and metal oxyhalide compounds have been shown as potential photocatalysts, *e.g.*, Bi_2_MO_4_Cl (M = Y/La/Bi), PbBiO_2_X, Bi_12_O_17_Cl_2_, Bi_3_O_4_Cl, Bi_24_O_31_Cl_*x*_Br_10−*x*_, PbBiO_2_Br/BiOBr, PbBiO_2_Br/UiO-66-NH_2_ (mpg-C_3_N_4_/PbBiO_2_Br), Bi_4_NbO_8_Cl, Bi_24_O_31_Cl_10_.^1,2,5,14–16^

Visible light responsive photocatalysis is responsible for the photocatalyzed decolorization of a dye in solution *via* a photoexcitation process.^17^ Despite substantial research endeavours in the field of visible light responsive photocatalysts during past few decades, development of such materials still remains a challenge for us. It is promising to see that a number of novel catalysts have been reported, such as Ce(IO_3_)_4_, CeGeO_4_, ZrHIO_6_·4H_2_O, CeHIO_6_·4H_2_O, Fe_2_O_3_–CeO_2_–TiO_2_/γ-Al_2_O_3_, Ce-doped MoO_3_, CuO–MoO_3_–P_2_O_5_, ZnO/MoO_3_/SiO_2_, Pt–HCa_2_Nb_3_O_10_, ZrGeO_4_, which have the ability to degrade organic dyes in the dark condition even at room temperature.^18–28^

Oxyhalides comprising p-block elements are classified as the members of the [L–O–X] family (L = p-element lone-pair cation, X = halide ion). Some well-known oxyhalides with group 15 cations (Sb^3+^, Bi^3+^), that have been reported to date are SbOCl,^29^ onoratoite,^30^ Sb_3_O_4_Cl,^31^ Sb_4_O_5_Cl_2_,^32,33^ Sb_8_O_11_Cl_2_,^34^ Bi_3_O_4_Br, BiOBr.^35^ Presence of both the halide ions and the electronic lone pair of the cations generate an interesting stereochemical environment in the oxyhalide (X = Cl/Br) compounds. They introduce a special channel or void space or a layered zone inside the crystal structures, where both the units are bound by weak van der Waals interactions.^35^ In these materials, chlorine and bromine are located between layers, formed by L cations and O anions.

In this work, we have prepared single crystals of both Sb_4_O_5_Br_2_ and Sb_4_O_5_Cl_2_ by hydrothermal technique. A series of both compounds were prepared by varying the pH level using the hydrothermal method. Structural studies of Sb_4_O_5_Br_2_ were previously performed by Maja Edstrand and coworkers by using ‘Patterson project’ in 1947, but the authors did not specify the nature of the antimony bonds and uncertainties associated with the positions of the oxygen atoms.^32^ In our study, for the first time, we have eliminated these disadvantages and presented a comprehensive structural analysis of compound Sb_4_O_5_Br_2_ using single-crystal X-ray diffraction data and a comparative study with Sb_4_O_5_Cl_2_ has also been discussed. Dye degradation reactions using both Sb_4_O_5_Br_2_ and Sb_4_O_5_Cl_2_ catalyst under both dark and sunlight conditions have also been investigated for the first time and a comparative study of the electronic band energies of the synthesized catalysts is also shown.

## Experimental section

### Synthesis

Single crystals of Sb_4_O_5_X_2_ (X = Cl/Br) were synthesized by hydrothermal technique. SbCl_3_ : Sb_2_O_3_ = 2 : 5 and SbBr_3_ : Sb_2_O_3_ = 2 : 5 mixtures were kept in two separate Teflon-lined steel autoclaves of 18 ml each. 3 ml of deionized water was added to each and stirred for half an hour using a magnetic stirrer. The autoclaves were heated to 230 °C. The plateau temperature was maintained for 4 days, and thereafter the temperature was lowered to 30 °C at a rate of 0.6 °C min^−1^. The schematic representation of the synthetic procedure was presented in the ESI as Fig. S1.† The following chemicals were used as starting materials: Sb_2_O_3_ (99.6%, Alfa Aesar), SbCl_3_ (99%, Merck) and SbBr_3_ (99%, Alfa Aesar). The off-white transparent crystals of Sb_4_O_5_Cl_2_ and Sb_4_O_5_Br_2_ were obtained, after being washed several times with water and ethanol, followed by drying at room temperature (Fig. S1†). The synthesis of both compounds was performed from pH 2 to 6.

### Characterization techniques

A Bruker D8 Venture diffractometer equipped with a PHOTON 100 detector was used to collect single crystal diffraction data. Oblique incidence correction and data integration were done by using the SAINT software package.^36^ Absorption correction was done using SADABS.^37^ Superflip^38^ program was used to solve crystal structures and refinement was done using JANA 2006.^39^ Anisotropic refinement of all atoms was performed as well.

A powder X-ray diffraction study was used to determine the phase purity of the prepared compounds, and the raw data were obtained using a Panalytical X'Pert PRO X-ray powder diffractometer in Bragg Brentano geometry with Cu Kα radiation (=1.54060 Å). A fast scanning mode was implemented in 2*θ*, which ranged from 4° to 70° with a step size of 0.0131°. Each observed reflection was indexed on the basis of the refined crystal structure obtained from the single-crystal data. The powder diffraction pattern was checked and refined using JANA 2006 and the data is well matched with the single-crystal structure refinement data (see Fig. S2 and S3†).

FTIR spectroscopic studies were carried out using a PerkinElmer L 120-000A spectrometer in the region of 4000–400 cm^−1^. Density Functional Theoretic (DFT) studies were carried out on the crystal structures of the two compounds with the LANL2DZ basis set^40,41^ and PBE density functional^42^ using the Gaussian 09W program suite.^43^ The vibration frequencies were calculated. These were fitted with a FORTRAN program and theoretical FTIR plots were generated.

Morphological characterization of the samples was performed by means of Field Emission Scanning Electron Microscope (FESEM), Zigma, Carl Zeiss, Germany, 30 kV, image resolution: 1.3 nm, energy resolution ∼127 eV. Powder samples have been dispersed in IPA medium (for 30 minutes) and dropped on a glass substrate, followed by drying under an IR lamp for SEM study.

Standard methyl orange (MO) was used for the dye degradation reactions, which were performed in both the absence and presence of sunlight. The synthesized catalysts were dissolved into the MO solution (10 ppm). The solution was stirred in the absence of light for hours until it reached adsorption equilibrium. Then they were irradiated by sunlight (UV irradiation source) with continuous stirring. During that time, solutions were collected and measured at 30 minutes intervals until degradation was completed. The decolorization reaction of catalyzed MO solution in both dark and light conditions was then monitored by a UV-Vis spectrophotometer. The optimal value for taking the amount of catalyst and the dye concentration was chosen after several experiments.

The catalysts were also dissolved in aqueous solution to compare the spectra against the MO-dye solution. The absorbance spectra of both the aqueous and dye solutions of each catalyst were studied by the spectrophotometer by changing the wavelength from 200 nm to 900 nm, from which *λ*_max_ values were obtained in each case. The absorbance values were measured for each solution at fixed *λ*_max_ value for each case obtained previously. The reaction was started at room temperature and after complete degradation in presence of sunlight, the temperature turned to around 35 °C.

## Result and discussion

### Crystal structure

**Fig. 1:**
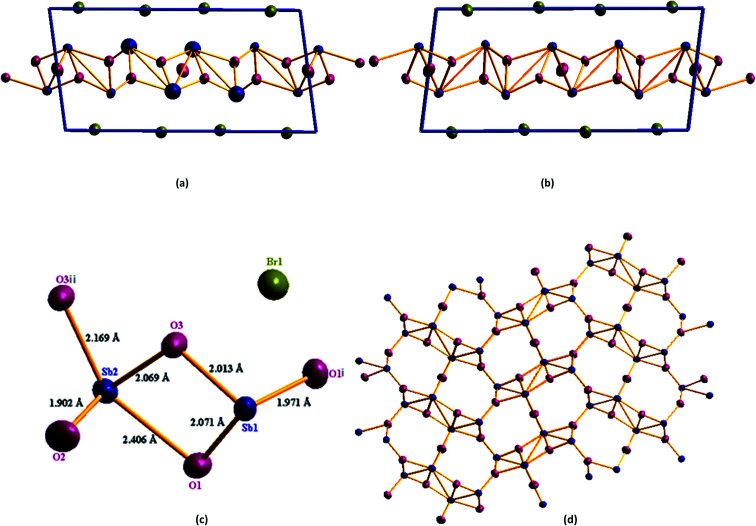
(a) Unit cell crystal structure of Sb_4_O_5_Br_2_, (b) unit cell crystal structure of Sb_4_O_5_Cl_2_. (c) Asymmetric unit of Sb_4_O_5_Br_2_, symmetry operation for O1i and O3ii are (1 − *x*, −0.5 + *y*, 0.5 − *z*) and (*x*, 1.5 − *y*, 0.5 + *z*) respectively. (d) Antimony–oxygen cage structure, here blue represent Sb atom, pink represent O atom and green represent Br atom.

Single crystal X-ray diffraction study shows that Sb_4_O_5_Br_2_ crystallizes in the monoclinic symmetry (*P*2_1_/*c*) with the unit cell parameters *a* = 6.60739(9) Å, *b* = 5.14333(6) Å, *c* = 13.46775(17) Å, *α* = 90.0°, *β* = 97.9092(13)°, *γ* = 90.0° and *Z* = 2. The crystallographic parameters are summarized in Table 1. The asymmetric unit of this compound consists of two antimony atoms, three oxygen atoms and one bromine atom and the BVS calculation supports the oxidation number of each antimony atom as +3, oxygen atoms as −2 and bromine atom as −1 respectively (Table S1†). Sb1 coordinated with three oxygen atoms to form trigonal pyramidal [SbO_3_] unit and Sb2 coordinated with four oxygen atoms to form trigonal bipyramidal [SbO_4_] unit. [Sb(1)O_3_] and [Sb(2)O_4_] are connected by an edge sharing Sb(1)–O–Sb(2) bond to form the [Sb_2_O_5_] unit. Two [Sb_2_O_5_] units are connected by corner sharing Sb–O–Sb to form infinite chain of [(Sb_4_O_5_)^2+^]_*n*_ unit (Fig. 1).

**Table 1:** Crystallographic data for Sb_4_O_5_Br_2_ and Sb_4_O_5_Cl_2_

Chemical formula	Sb_4_O_5_Br_2_	Sb_4_O_5_Cl_2_	Sb_4_O_5_Cl_2_ (ref. 33)
Formula weight/g mol^−1^	726.82	637.90	637.90
Temperature/K	293	293	—
Crystal system	Monoclinic	Monoclinic	Monoclinic
Space group	*P*2_1_/*c* (14)	*P*2_1_/*c* (14)	*P*2_1_/*c* (14)
*a*/Å	6.6073(10)	6.2435(3)	6.2393(8)
*b*/Å	5.1428(10)	5.11440(10)	5.1132(7)
*c*/Å	13.4696(2)	13.5502(6)	13.5351(18)
*β*/deg	97.9328(11)	97.252(3)	97.14(2)
*V*/Å^3^	453.317(13)	429.22(3)	428.43(10)
*ρ*/g cm^−3^	5.3247	4.9357	4.495
*Z*	2	2	2
Wavelength/Å	0.71073	0.71073	—
Indices range	−14 ≤ *h* ≤ 13	−12 ≤ *h* ≤ 12	—
	−10 ≤ *k* ≤ 10	−10 ≤ *k* ≤ 10	
	−28 ≤ *l* ≤ 28	−26 ≤ *l* ≤ 26	
No. of reflections measured/unique	35 836/4611	35 050/3541	2293/929
*R*_int_	2.42	3.41	—
(sin *θ*/*λ*)max/Å^−1^	0.8	0.8	—
*R*(obs)/*wR*(all)	2.42/2.91	3.24/3.63	5.38/1.47
Δ*ρ*_max_/Δ*ρ*_min_	0.70/−0.68	3.22/−1.45	—
GOF(obs)/GOF(all)	1.49/1.41	1.44/1.28	—

Table 2 summarizes all Sb–O distances found in [(Sb_4_O_5_)^2+^]_*n*_ units. The Br^−^ ions are located between the [(Sb_4_O_5_)^2+^]_*n*_ layers. There is no direct covalent bond between antimony and the halide atom (Br1) as the bond length is more than 3 Å, whereas, the normal covalent bond length of Sb–Br is in the range of 2.46–2.54 Å.^44^ A comprehensive structural analysis has been performed in this study and repaired the uncertainties over the atomic positions mentioned by Maja Edstrand in 1947.^32^

**Table 2:** Atomic distances for Sb_4_O_5_Br_2_

Atoms	Oxygens	Distance (Å)
Sb1	O1	1.963(3)
	O1	2.059(3)
	O3	2.011(3)
Sb2	O1	2.460(3)
	O2	1.8997(2)
	O3	2.079(2)
	O3	2.145(2)

The study also shows more precise structural parameters of Sb_4_O_5_Cl_2_ than the previously reported antimony oxychloride compound (see Table 1).^32,33^ The lower *R* value for the model obtained from our experiment is also indicative of a better structural model. When Sb_4_O_5_Br_2_ is compared to Sb_4_O_5_Cl_2_, slight elongation along the *a* and *b* axes is observed in Sb_4_O_5_Br_2_, whereas slight elongation along the *c* axis is observed in Sb_4_O_5_Cl_2_. This result is well suited to the larger Sb–Br bond distance (3.004 Å) compared to the Sb–Cl bond distance (2.94 Å), which means the van der Waals gap is higher in between [Sb_4_O_5_^2+^] layers in Sb_4_O_5_Br_2_. The lone pairs present in Sb (1) and Sb (2) are directed away from the layer. Corresponding Sb–O bond lengths for antimony oxy-chloride are listed in Table 3. The Sb–O distances are found to be comparable with the distances found in cubic Sb_2_O_3_ and orthorhombic Sb_2_O_3_.^45^

**Table 3:** Atomic distances for Sb_4_O_5_Cl_2_

Atoms	Oxygens	Distance (Å)
Sb1	O1	1.9708(14)
	O1	2.0715(16)
	O3	2.0130(14)
Sb2	O1	2.4060(15)
	O2	1.90154(12)
	O3	2.0688(15)
	O3	2.1690(13)

### Vibrational spectral studies

**Fig. 2:**
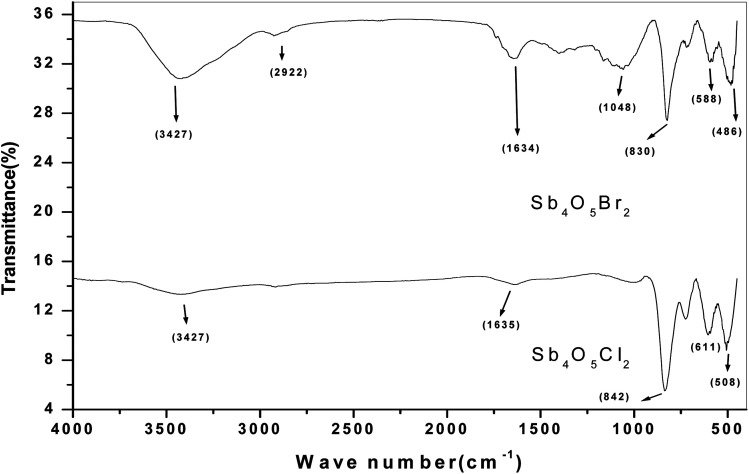
FTIR spectra of antimony oxychloride and antimony oxybromide.

**Fig. 3:**
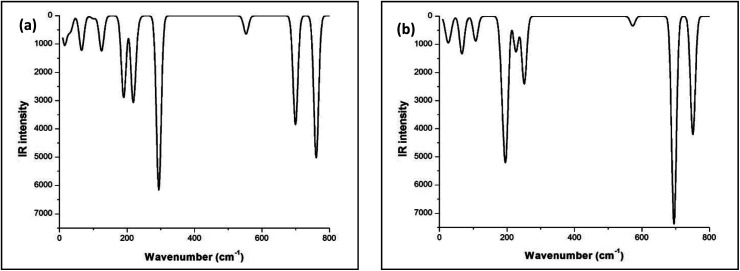
Theoretical FTIR curve for (a) Sb_4_O_5_Cl_2_ and (b) Sb_4_O_5_Br_2_.

The FTIR studies of both Sb_4_O_5_Br_2_ and Sb_4_O_5_Cl_2_ show strong peaks at 830 cm^−1^ and 842 cm^−1^ respectively, which are due to the Sb–O–Sb bond, and the signals around 500 cm^−1^ to 600 cm^−1^ are responsible for the Sb–O bonds are responsible for other. The broad peak at 3427 cm^−1^ is due to the presence of water (see Fig. 2). No covalency is formed in the case of Sb–Cl and Sb–Br bonds and hence no signal is responsible for the structure. The theoretical FTIR curve for Sb_4_O_5_Cl_2_ and Sb_4_O_5_Br_2_ is comparable with the experimental value in the IR region (see Fig. 3).

### Photocatalytic studies

**Fig. 4:**
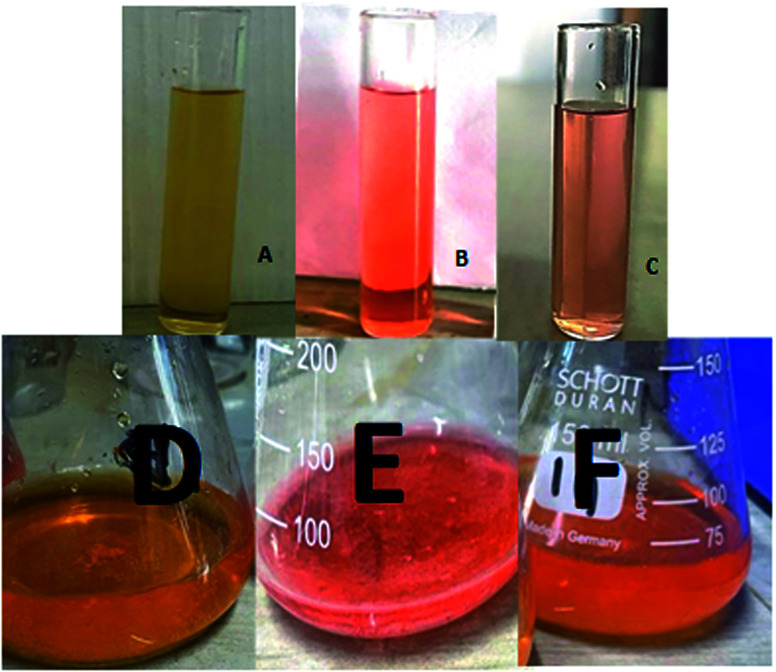
(A) Sb_4_O_5_Br_2_ catalyzed dye solution at *t* = 0 (B) *t* = 120 min (dark condition); (C) *t* = 240 min (sunlit condition) and (D) Sb_4_O_5_Cl_2_ catalyzed dye solution at *t* = 0 min, (E) *t* = 90 min (dark condition); (F) *t* = 210 min (sunlit condition).

**Fig. 5:**
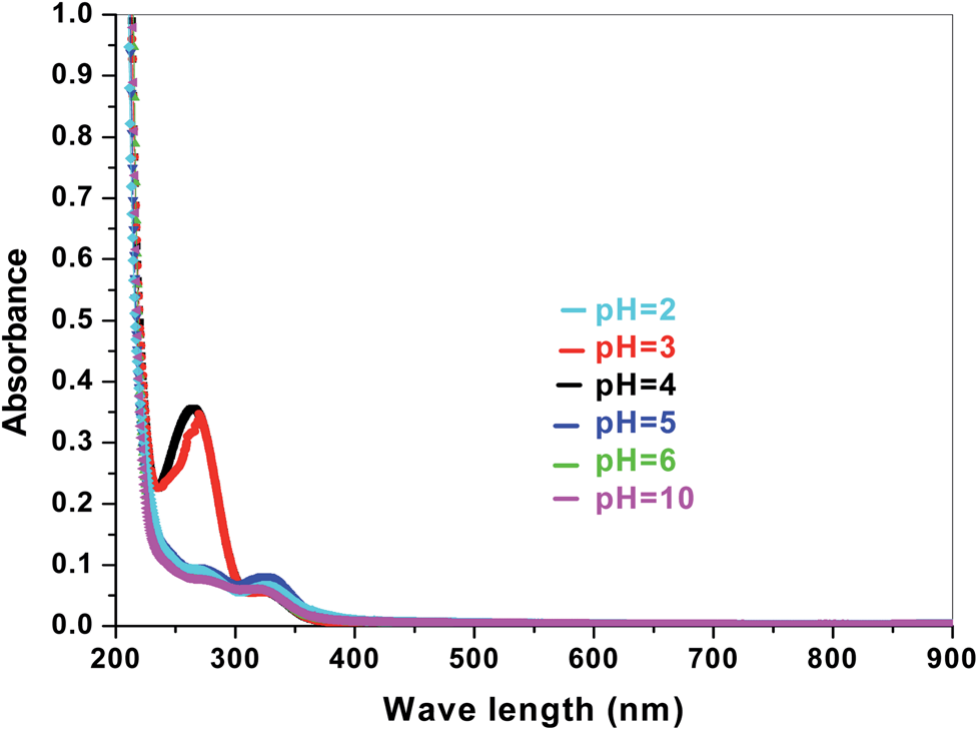
Absorption spectra for Sb_4_O_5_Br_2_ at various pH in aqueous solution.

**Fig. 6:**
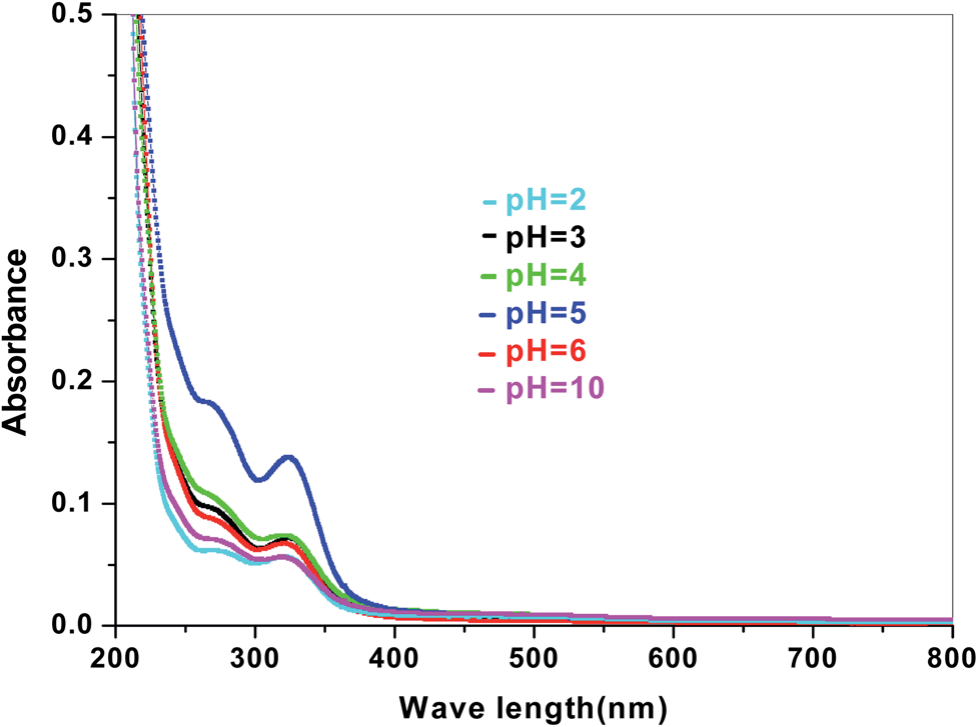
Absorption spectra for Sb_4_O_5_Cl_2_ at various pH in aqueous solution.

**Fig. 7:**
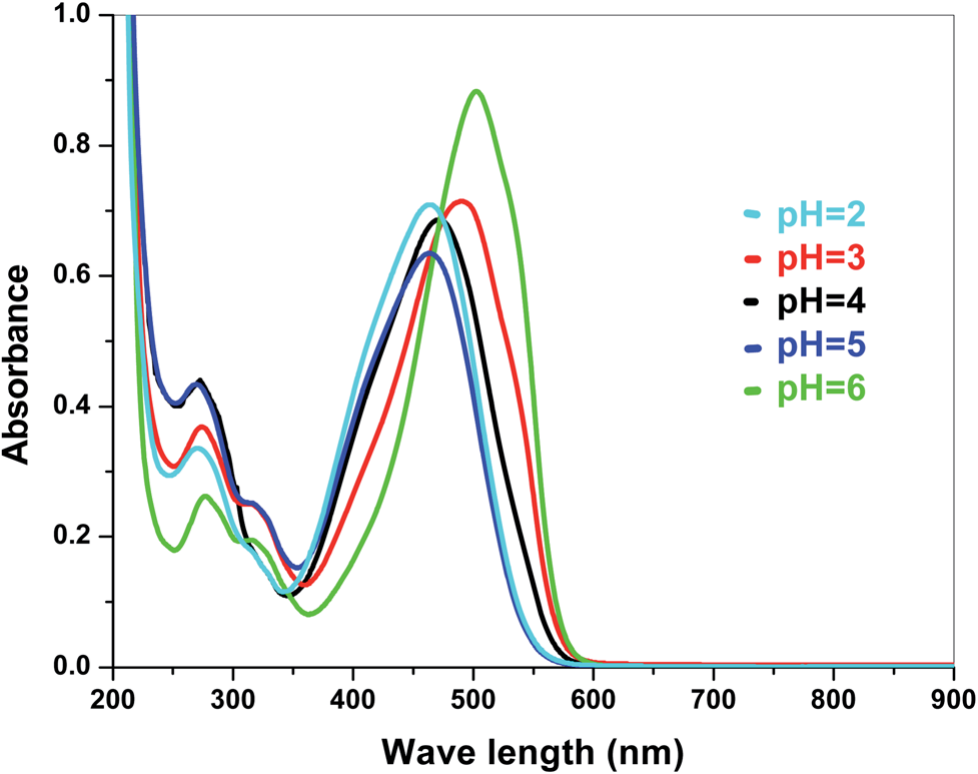
Absorbance spectra of Sb_4_O_5_Br_2_ in various pH in dye solution.

**Fig. 8:**
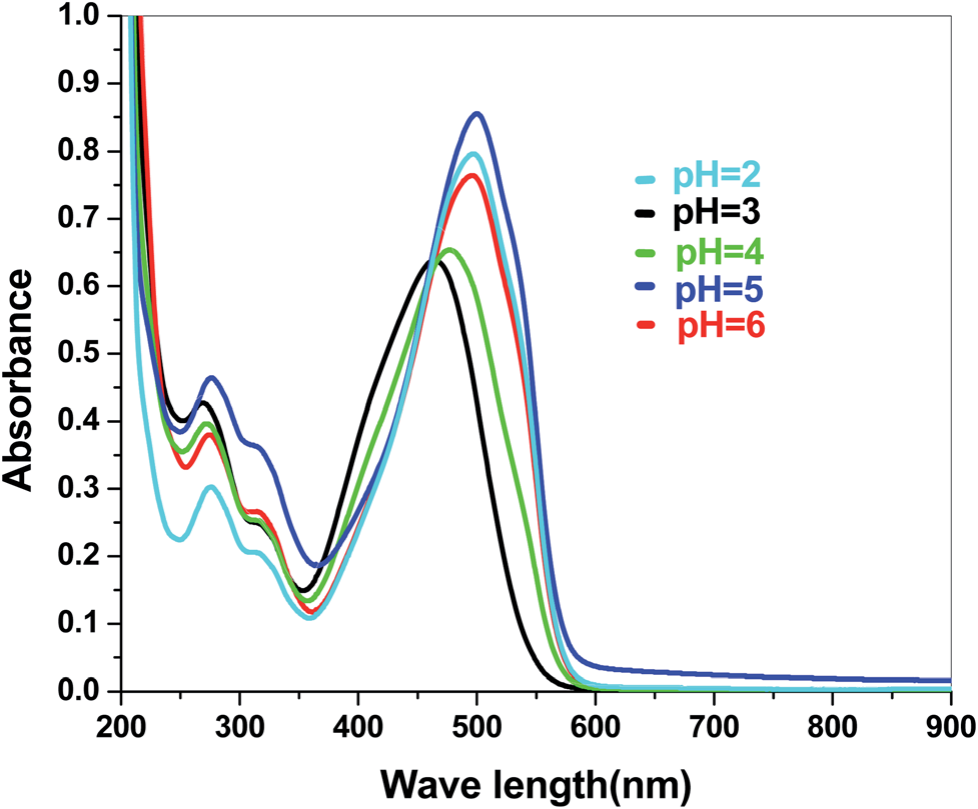
Absorbance spectra of Sb_4_O_5_Cl_2_ in various pH in dye solution.

**Fig. 9:**
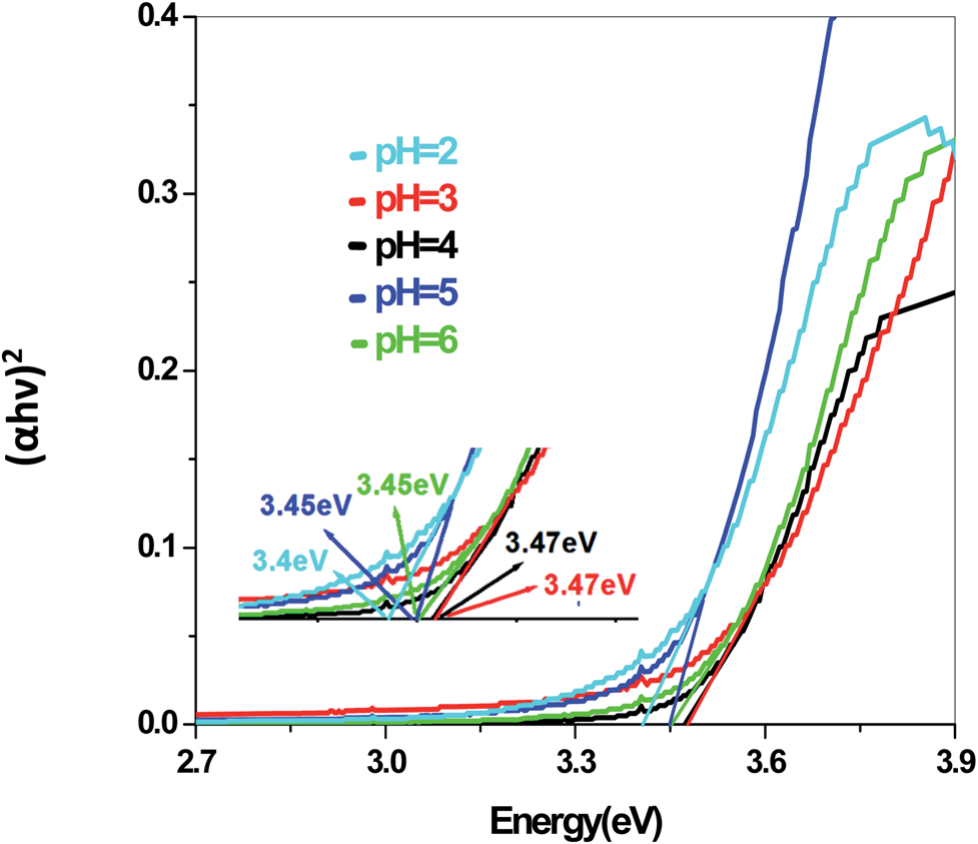
Band gap plot of Sb_4_O_5_Br_2_ in aqueous solution.

**Fig. 10:**
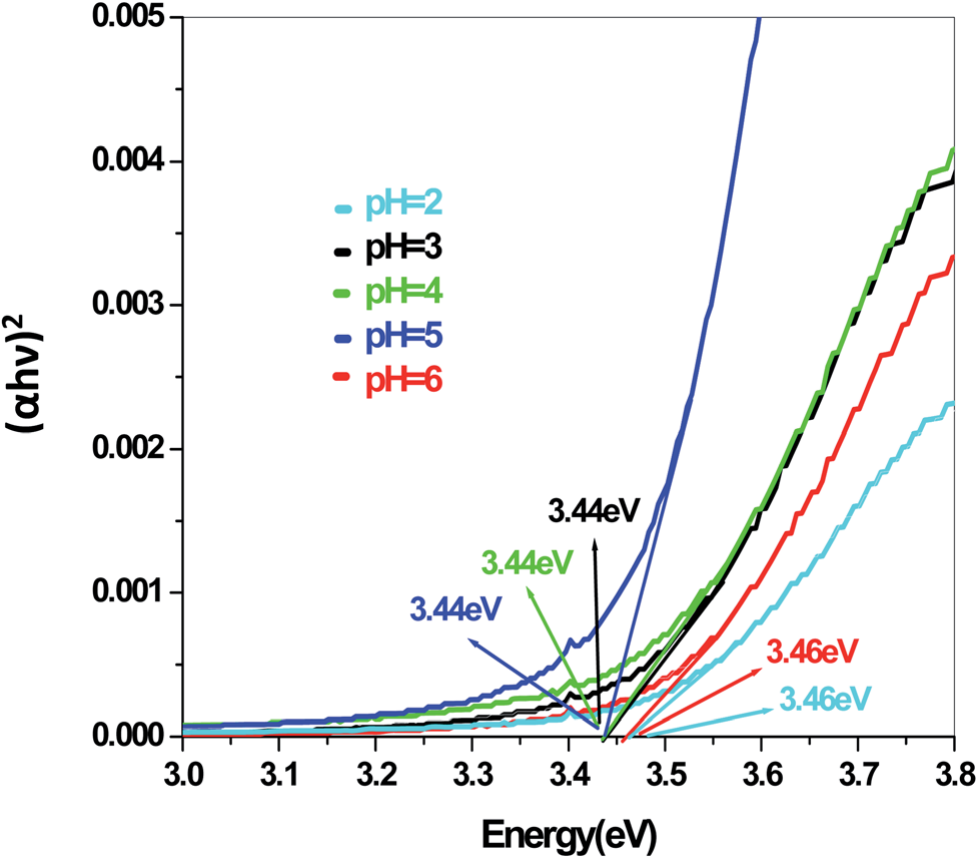
Band gap plot for Sb_4_O_5_Cl_2_ in aqueous solution.

**Fig. 11:**
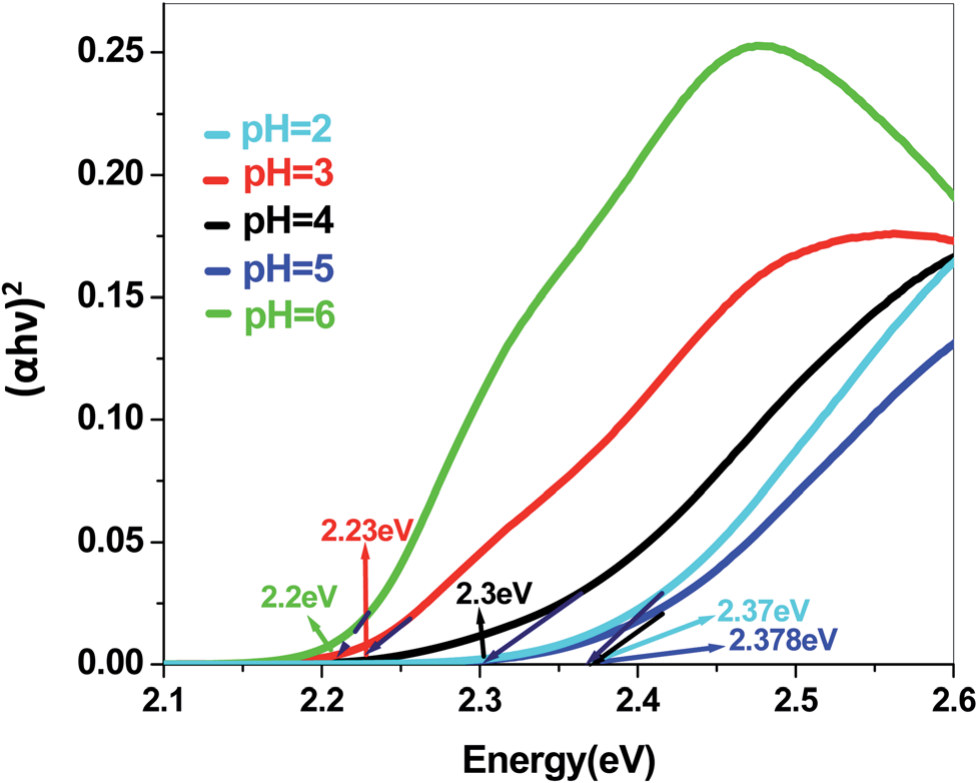
Band gap plot of Sb_4_O_5_Br_2_ in dye solution.

**Fig. 12:**
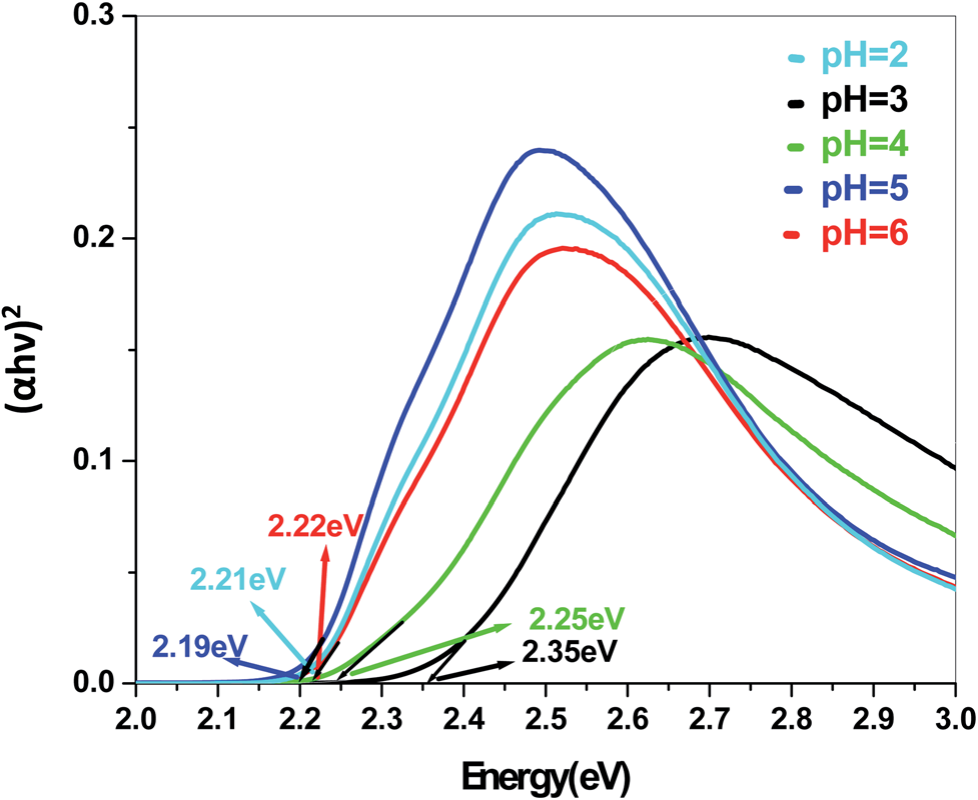
Band gap plot for Sb_4_O_5_Cl_2_ in dye solution.

**Fig. 13:**
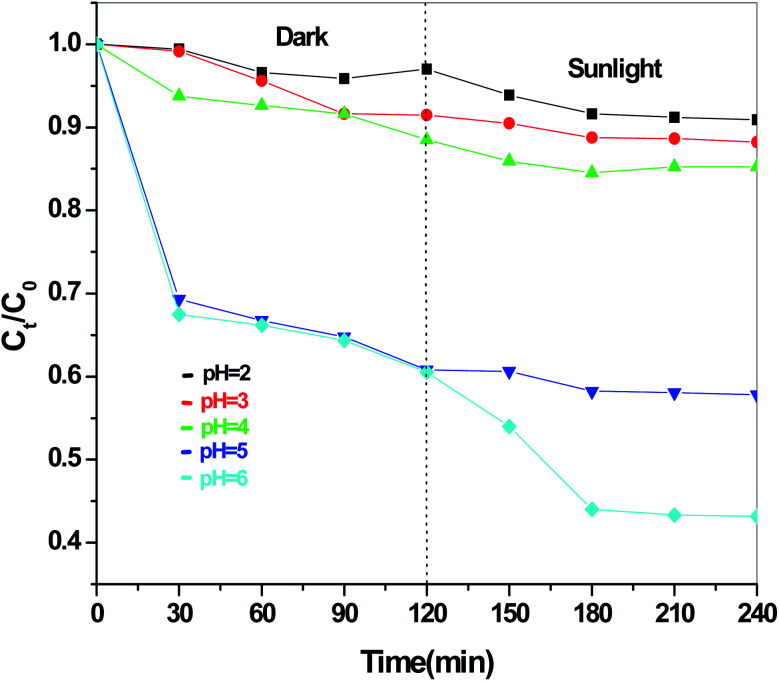
Concentration (*C*_*t*_/*C*_0_) *vs.* reaction time (min) graph for Sb_4_O_5_Br_2_ at different pH.

**Fig. 14:**
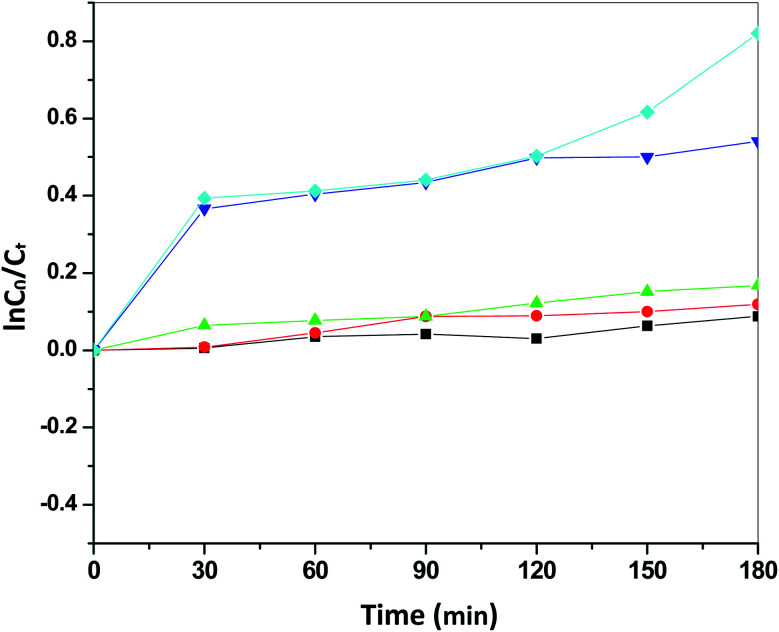
ln(*C*_0_/*C*_*t*_) *vs.* reaction time over Sb_4_O_5_Br_2_ samples prepared at different pH.

**Fig. 15:**
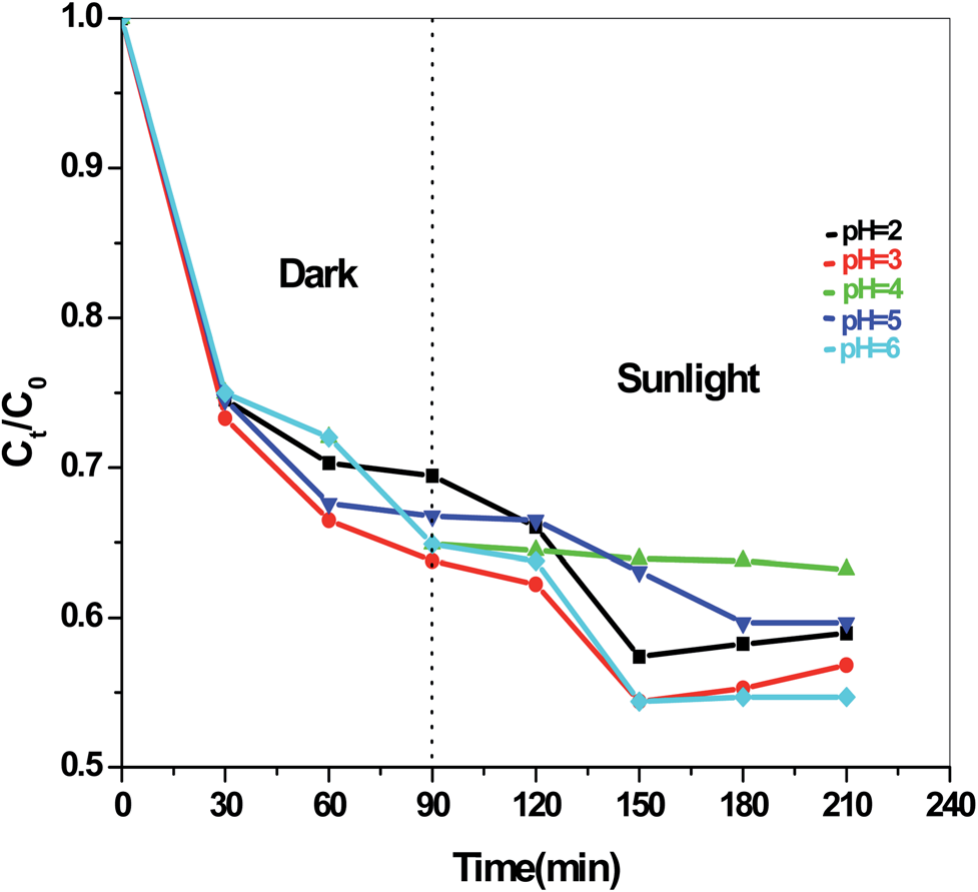
Concentration (*C*_*t*_/*C*_0_) *vs.* reaction time (min) graph for Sb_4_O_5_Cl_2_ at different pH.

**Fig. 16:**
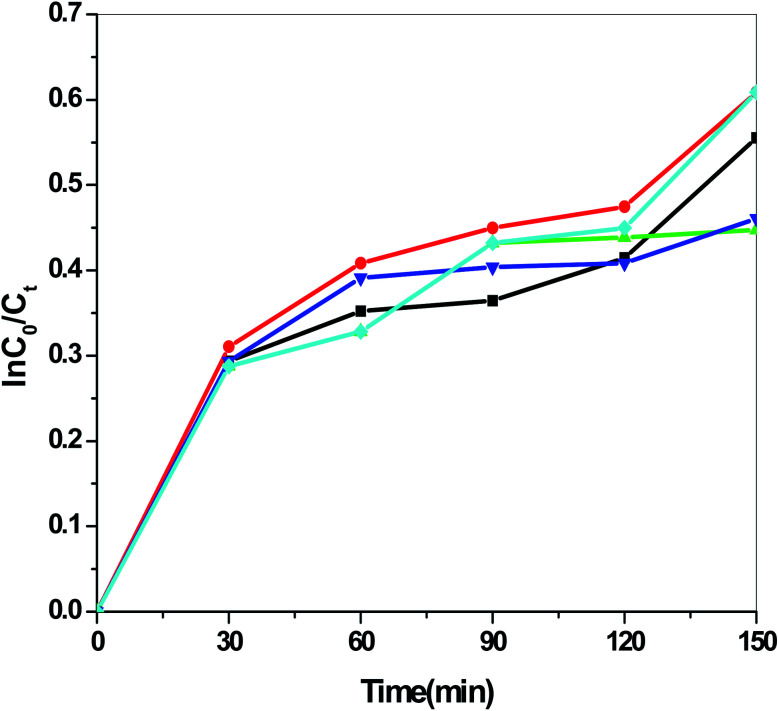
ln(*C*_0_/*C*_*t*_) *vs.* reaction time over Sb_4_O_5_Cl_2_ samples prepared at different pH.

The dye degradation efficiencies of Sb_4_O_5_Br_2_ and Sb_4_O_5_Cl_2_ are different from each other. Maximum dye degradation was achieved at pH = 6 and pH = 5 at 59% and 45%, respectively, while the Sb_4_O_5_Br_2_ sample produced only 5% to 16% dye degradation at lower pH (pH = 2–4). An interesting fact is that about 40% and 42% of dye decomposition in the dark is achieved at pH = 5 and pH = 6, respectively, and the amount gradually decreases at lower pH. At pH = 4, the degradation in the dark is reduced by a factor of two, and at pH = 2, it is less than 5%. In the case of Sb_4_O_5_Cl_2_, 38% to 48% total dye degradation was observed, with a degradation of approximately 33% to 36% in the dark at various pH ranges from 2 to 6. The photocatalytic activity of Sb_4_O_5_Cl_2_ under light irradiation has been previously observed,^11^ but so far no such dye degradation has been reported in both dark and light conditions. Fig. 4 shows the color change during degradation in both dark and sunlight. Fig. 5–16 shows the behavior of MO decoloration of Sb_4_O_5_Br_2_ and Sb_4_O_5_Cl_2_ under sunlight.

The electronic band structures of the synthesized crystallite samples Sb_4_O_5_Br_2_ and Sb_4_O_5_Cl_2_ are obtained in both aqueous and dye solution, were analyzed by UV-Vis absorption spectra and Tauc plots are also shown. In this case, the corresponding *λ*_max_ was obtained at around 364 nm in the aqueous system for both compounds, but in the dye solution it varied with the change of pH. The absorption spectra of the samples are shown in Fig. 5, 6, 7 and 8 respectively. For the Sb_4_O_5_Br_2_ samples, the *λ*_max_ values are more red shifted, showing highest *λ*_max_ for pH = 6 at about 500 nm, while in case of the Sb_4_O_5_Cl_2_, it shows almost similar *λ*_max_ (∼500 nm) at pH = 2,5,6, whereas at pH = 3 and 4, *λ*_max_ is obtained at 465 nm and 476 nm, respectivevly.

In a conventional semiconductor, the direct band gap energies can be calculated by the Tauc equation:(*αhν*)^2^ = *A*(*hν* − *E*_g_)where *α*, *ν*, *A* and *E*_g_ are absorption coefficient, light frequency, proportionality constant and band gap energy respectively. The band gap energies of the samples prepared at pH = 2, 3, 4, 5 and 6 are summarized in the ESI (Tables S2 and S3†), as obtained from the intercept of the tangents to the plots shown in Fig. 9, 10, 11 and 12.

Fig. 13 presents the plot of the concentration (*C*_*t*_/*C*_0_) of MO solution *versus* the reaction time (*t*) of Sb_4_O_5_Br_2_ samples prepared at pH = 2, 3, 4, 5, 6 respectively. This degradation process of MO is in accordance with the first-order kinetics model of the Langmuir–Hinshelwood equation, and is expressed as:ln(*C*_0_/*C*_*t*_) = *kt* + *x*,where *k* is the apparent reaction rate constant, *C*_0_ is the initial concentration of aqueous MO, *t* is the reaction time, and *C*_*t*_ is the concentration of aqueous MO at time ‘*t*’. The calculated reaction rate constants (*k*) are 0.00156, 7.491 × 10^−4^, 9.394 × 10^−4^, 7.580 × 10^−4^ and 9.174 × 10^−4^ min^−1^ for the intercept (*x*) = 0.00681, 0.00226, 0.054, 0.00185 and 0.117 at pH = 2, 3, 4, 5, 6 respectively as shown in Fig. 14. The concentration of MO (*C*_*t*_/*C*_0_) solution as a function of the reaction time (min) of the Sb_4_O_5_Cl_2_ samples, prepared at pH = 2, 3, 4, 5, 6 respectively, is shown in Fig. 15. For Sb_4_O_5_Cl_2_, calculated rate constants are 0.00222, 0.00238, 0.00174, 0.00196, 0.00264 min^−1^ for *x* = 0.14801, 0.17649, 0.1726, 0.16787, 0.13657 for pH = 2, 3, 4, 5, 6 respectively (see Fig. 16).

The energy gap is very similar for both Sb_4_O_5_Br_2_ and Sb_4_O_5_Cl_2_, which is about 3.4 eV, obtained from the aqueous solutions. It varies largely when the dye solution of MO with synthesized catalysts is used and lowered to about 2.2–2.3 eV for both compounds. This variation may be due to interference from MO dye solution itself, since the values of the absorption coefficient, in this case, are the sum of the spectra of both the MO dye and the Sb_4_O_5_X_2_ compounds. Therefore, it can be concluded that to obtain the accurate band gap values, only aqueous solutions should be used, which can be the characteristic bandgap for the semiconductor itself.^46^ Yang *et al.* also studied the band gaps for the Sb_4_O_5_Cl_2_ compound at different pH and the value is about 3.3 eV.^11^

The band gap values are also well matched with the computational study carried out by Ran *et al.*^47^ The band gap energies of Sb_4_O_5_Br_2_ compounds vary from 3.4 eV to 3.47 eV with the change of pH (Fig. 9). A relatively slight smaller band gap has been found at pH = 2 (3.4 eV). These compounds may be promising as solar absorbers in the UV region.^11,47^

**Fig. 17:**
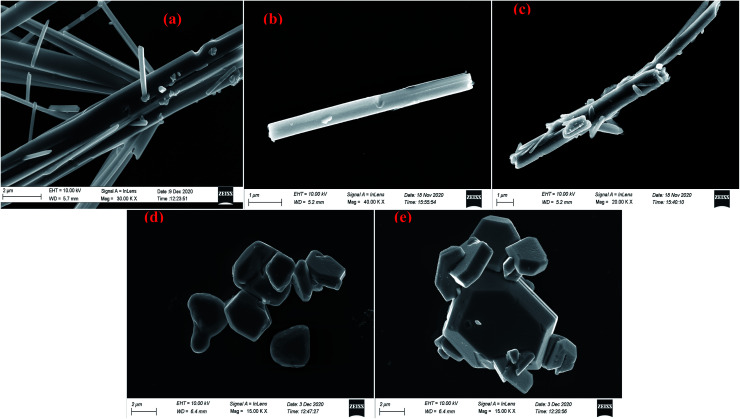
SEM images of antimony oxybromide at (a) pH = 2, (b) pH = 3, (c) pH = 4, (d) pH = 5, (e) pH = 6.

**Fig. 18:**
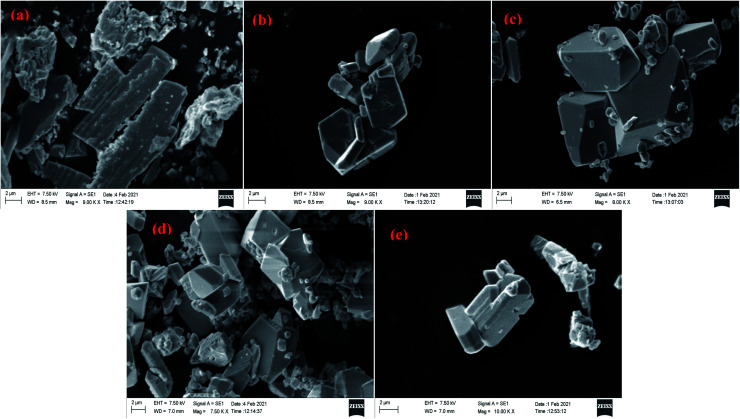
SEM images of antimony oxychloride at (a) pH = 2, (b) pH = 3, (c) pH = 4, (d) pH = 5, (e) pH = 6.

Scanning electron microscopic study reveals that needle shaped particles at pH = 2, 3 transform into hexagonal cluster shaped particles at pH = 5, 6 while both needle and clusters of hexagons are observed at pH = 4. Henceforth, it can be concluded that the surface morphology of the products is transformed well in accordance with the acidity of precursor solutions (Fig. 17 and 18). However, no such morphological changes were observed for Sb_4_O_5_Cl_2_ by changing the pH. The shape and size of the particles of both Sb_4_O_5_Br_2_ and Sb_4_O_5_Cl_2_ have been listed in ESI (Table S4†).

Yang *et al.* also reported a slight change in surface morphology due to the change of pH of hydrothermally synthesized Sb_4_O_5_Cl_2_ crystallite (160 °C for 12 h).^11^ It showed hollow sphere shape with irregular cuboids at pH = 2 and it transforms into microbelt like particles at higher pH.

From the above study, it could be suggested that the compounds with large surface areas effectively adsorb the dye molecules, which leads to the rupture of labile azo bonds of the methyl orange dye and furnishes free electrons that may cause the dye degradation for certain compounds at dark conditions, as those electrons have the capability to form reactive oxygen species (ROS), *i.e.*, superoxide radical anions and hydroxyl radicals.^20,48,49^ However, due to the absence of conducting metals in the synthesized compounds, only partial dye degradation could be achieved in dark conditions. Furthermore, the solution is subjected to solar light illumination after it attains adsorption–desorption equilibrium, and as a result, ROS get produced again and causes further dye degradation.^1,11,50^ Although complete degradation is not possible, which could probably due to the formation of some by-products. The probable mechanism of dye degradation in both dark and light condition is proposed as follows:

In dark condition:Sb_4_O_5_X_2_ + MO → Sb_4_O_5_X_2_ (MO)Sb_4_O_5_X_2_ (MO) ↔ Sb_4_O_5_X_2_ + MO^+^ + e^−^e^−^ + O_2_ → ˙O_2_^−^˙O_2_^−^ + H^+^ → HO_2_˙2HO_2_˙ → H_2_O_2_ + O_2_H_2_O_2_ + e^−^ → HO˙ + HO^−^HO˙ + MO/MO^+^ → … → by-products

In presence of sunlight:Sb_4_O_5_X_2_ + *hν* → Sb_4_O_5_X_2_ (e_CB_^−^ + h_VB_^+^)HO^−^ + h_VB_^+^ → HO˙H_2_O + h_VB_^+^ → HO˙ + H^+^e_CB_^−^ + O_2_ → ˙O_2_^−^˙O_2_^−^ + H^+^ → HO_2_˙2HO_2_˙ → ˙H_2_O_2_ + O_2_H_2_O_2_ + e^−^ → HO˙ + HO^−^HO˙ + MO → CO_2_ + H_2_O + by-products

## Conclusion

Sb_4_O_5_Br_2_ and Sb_4_O_5_Cl_2_ were synthesized by hydrothermal technique at 230 °C. The compounds belong to monoclinic symmetry with *P*2_1_/*c* space group, and lattice parameters are *a* = 6.6073(10), *b* = 5.1428(10), *c* = 13.4696(2), *β* = 97.9328° (11) and *a* = 6.2435(3), *b* = 5.11440(10), *c* = 13.5502(6), *β* = 97.252° (3), respectively. Small differences between crystal structures of bromide and chloride compounds have been observed. This is possibly because there is no direct bonding between (Sb_4_O_5_^2+^)_*n*_ units and halide atoms, which are present in the van der Waals gap. The distance between Sb^3+^ and halide atoms is greater in Sb_4_O_5_Br_2_ compared to Sb_4_O_5_Cl_2_, which can be attributed to differences of the size in Br and Cl.

Scanning electron microscopic studies reveal that needle shaped particles transform into hexagonal cluster shaped particles by increasing the pH for Sb_4_O_5_Br_2_, although no such morphological changes were observed for Sb_4_O_5_Cl_2_ by changing the pH. A comparative study to check the dye degradation (MO) using both compounds confirmed that the Sb_4_O_5_Br_2_ samples showed better (approximately 59%) degradation overall (both under dark and light conditions) at pH = 6 and it reduces up to 6% by lowering the pH. However, the Sb_4_O_5_Cl_2_ catalytic dye degradation was less dependent on pH, as it varied from 36% to 45% (both under dark and light conditions) under different acidic conditions, which is well justified to their SEM analysis because of the morphology remained the same upon pH change.

The band gap obtained in aqueous solution using Sb_4_O_5_Br_2_ samples is around ∼3.4 eV, while it varies from 2.2 to 2.37 eV in MO dye solution. Similarly, the energy gaps calculated from aqueous and dye solutions of Sb_4_O_5_Cl_2_ samples are around 3.4 eV and 2.19–2.35 eV, respectively. The electronic band structures, calculated using the aqueous solution of the prepared compounds, reveals wide band gap nature for both synthesized semiconductors. Due to the interference of the dye molecules, the dye mixed aqueous solution of the synthesized catalyst provides lower band gaps than the aqueous solution of the dye-free synthesized catalyst. Therefore, the accurate band gap values can be estimated only from the aqueous solutions of the catalyst, which can be considered as the characteristic bandgap of the semiconductor itself.

## Conflicts of interest

The authors have declared no conflict of interest.

## Supplementary Material

RA-012-D2RA01215D-s001

RA-012-D2RA01215D-s002
