# Supplementary material for: pH-regulated hydrothermal synthesis and characterization of Sb4O5X2 (X = Br/Cl) and its use for the dye degradation of methyl orange both with and without light illumination
Source: RSC Adv. 2022 Mar 15;12(14):8374–84. doi: 10.1039/d2ra01215d (PMC8984917; doi:10.1039/d2ra01215d)
Supplement: RA-012-D2RA01215D-s001 [file RA-012-D2RA01215D-s001.pdf]

Electronic Supplementary Material (ESI) for RSC Advances.
This journal is © The Royal Society of Chemistry 2022

pH-regulated hydrothermal synthesis, characterization of $Sb_4O_5X_2$ (X=Br / Cl) and its use for the dye degradation of methyl orange both with and without light illumination

Sayantani Paul[a], Bibaswan Sen[a], Nirman Chakraborty[b], Sangita Das[a], Swastik Mondal[b], Asoke P. Chattopadhyay[a], Sk Imran Ali[a*]

a. Department of Chemistry, University of Kalyani, Nadia, West Bengal, India

b. CSIR-Central Glass and Ceramic Research Institute, Jadavpur, Kolkata, West Bengal India.

Table S1. Bond Valence Sum Calculations for Sb4O5Cl2 and Sb4O5Br2 employing R_0(Sb–O) = 1.973Å ,R_0(Sb–Br) =2.50 Å, R_0(Sb–Cl)=2.35 Å

ATOMS	BVS
**Sb1**	2.5
**Sb2**	2.9
**Br**	0.7

ATOMS	BVS
**Sb1**	2.6
**Sb2**	2.7
**Cl**	0.6

Table S2: λ_{max}(nm) and band gap(eV) at different pH in aqueous solution:

pH	λ_{max} for $Sb_4O_5Cl_2$ (nm)	λ_{max} for $Sb_4O_5Br_2$ (nm)	E_g for $Sb_4O_5Cl_2$ (eV)	E_g for $Sb_4O_5Br_2$ (eV)
2	322	274.7	3.48	3.39
3	322	269.7	3.4	3.47
4	322	262.3	3.4	3.47
5	322	274.0	3.4	3.46
6	322	269.0	3.45	3.47

Table S3: λ_{max}(nm) and band gap (eV) at different pH in dye solution :

pH	λ_{max} for $Sb_4O_5Cl_2$ (nm)	λ_{max} for $Sb_4O_5Br_2$ (nm)	E_g for $Sb_4O_5Cl_2$ (eV)	E_g for $Sb_4O_5Br_2$ (eV)
2	496	462.9	2.215	2.37
3	462.4	489.5	2.356	2.30
4	476	471	2.247	2.20
5	500	462.5	2.198	2.376
6	496	502.7	2.215	2.22

Table S4: Shape and size of antimony oxybromide and antimony oxychloride :

pH	For $Sb_4O_5Br_2$		For $Sb_4O_5Cl_2$	
	Shape	Area (μm^2)	Shape	Area(μm^2)
2	Needle shaped	6.95×0.8	3D Sheet	24.75×15.40
3	Needle shaped	6.34×10.504	3D Sheet	14.375×6.075
4	Both needle and hexagonal shaped cluster	8.078× 7.09	3D Sheet	20.83×10.74
5	Hexagonal shaped cluster	4.669×3.748	3D Sheet	15.98×7.114
6	Hexagonal shaped cluster	17.244×0.458	3D Sheet	6.005×4.922

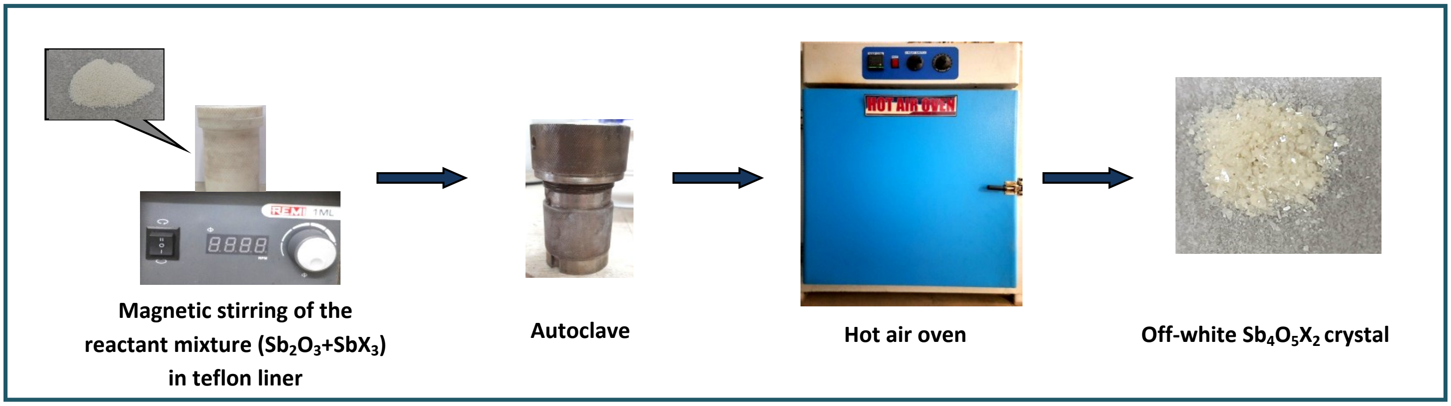

Fig S1: Schematic representation of experimental procedure of $Sb_4O_5X_2$ compounds

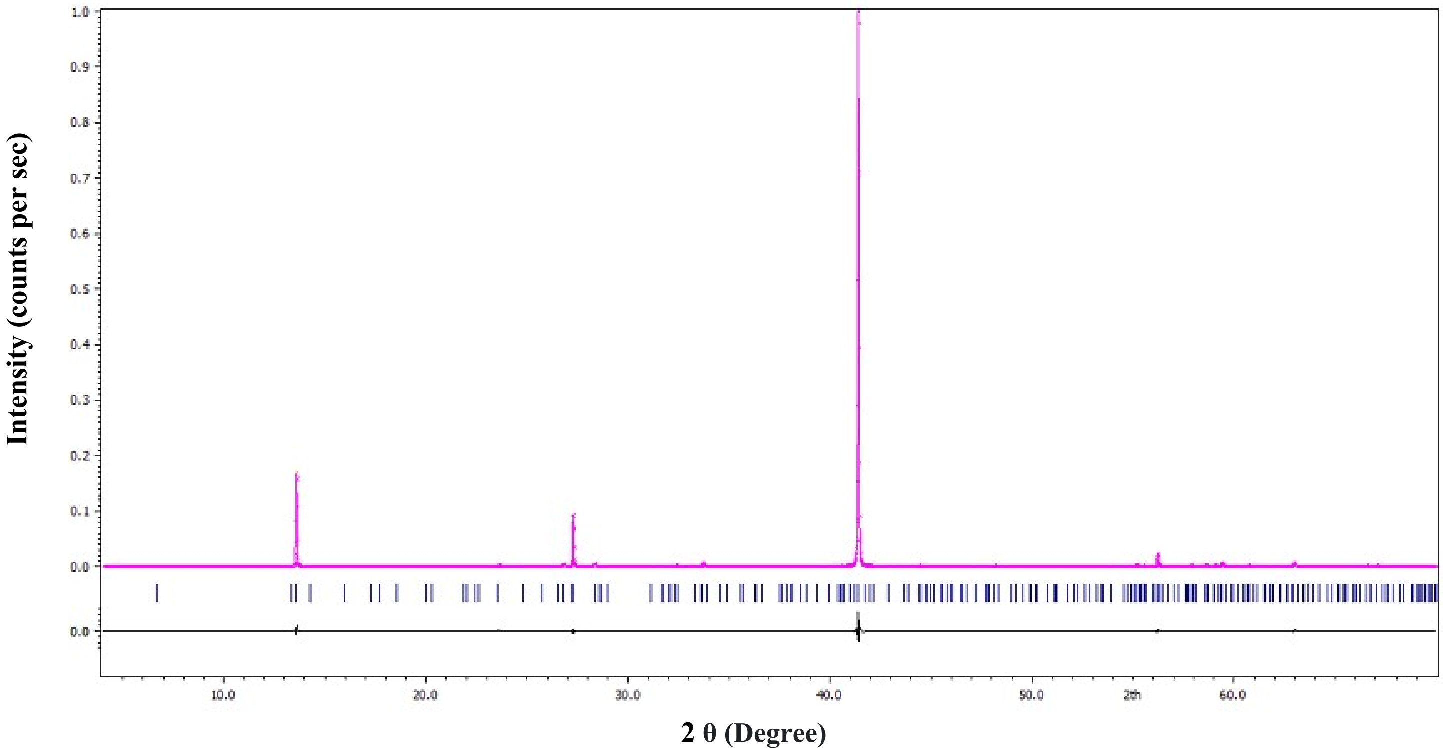

Fig S2: PXRD graph for $Sb_4O_5Br_2$

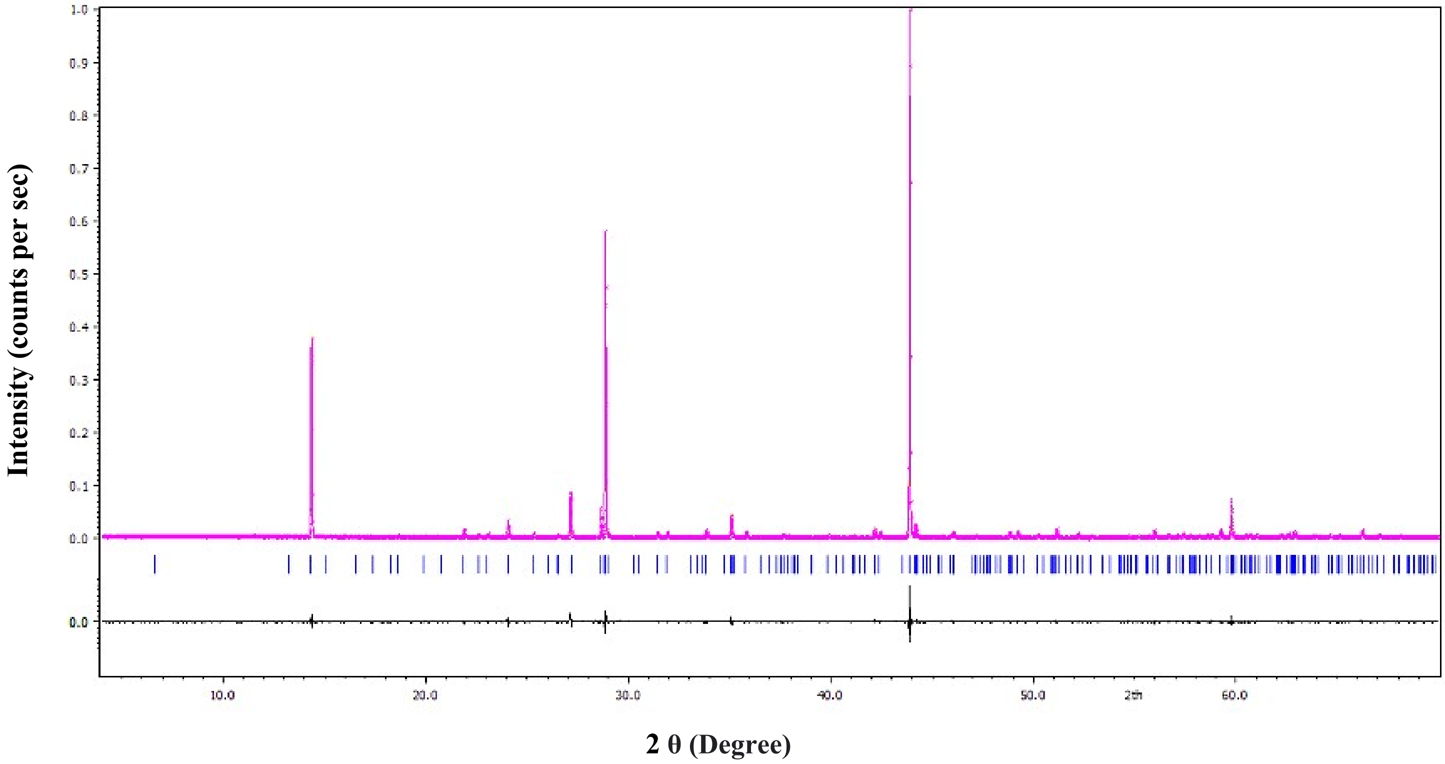

Fig S3: PXRD graph for $Sb_4O_5Cl_2$
